# Cytotoxic Activity of LLO Y406A Is Targeted to the Plasma Membrane of Cancer Urothelial Cells

**DOI:** 10.3390/ijms22073305

**Published:** 2021-03-24

**Authors:** Nataša Resnik, Larisa Tratnjek, Mateja Erdani Kreft, Matic Kisovec, Saša Aden, Apolonija Bedina Zavec, Gregor Anderluh, Marjetka Podobnik, Peter Veranič

**Affiliations:** 1 Institute of Cell Biology, Faculty of Medicine, University of Ljubljana, SI-1000 Ljubljana, Slovenia; natasa.resnik@mf.uni-lj.si (N.R.); larisa.tratnjek@mf.uni-lj.si (L.T.); mateja.erdani@mf.uni-lj.si (M.E.K.); 2 Department of Molecular Biology and Nanobiotechnology, National Institute of Chemistry, SI-1000 Ljubljana, Slovenia; matic.kisovec@ki.si (M.K.); sasa.aden@ki.si (S.A.); polona.bedina@ki.si (A.B.Z.); gregor.anderluh@ki.si (G.A.); marjetka.podobnik@ki.si (M.P.)

**Keywords:** bladder cancer, cancer urothelial cells, normal urothelial cells, listeriolysin O, blebbing, calcium influx, pore-forming toxin, cytolysin, cytotoxicity

## Abstract

Identification of novel agents for bladder cancer treatment is highly desirable due to the high incidence of tumor recurrence and the risk of progression to muscle-invasive disease. The key feature of the cholesterol-dependent toxin listeriolysin O mutant (LLO Y406A) is its preferential activity at pH 5.7, which could be exploited either directly for selective targeting of cancer cells or the release of accumulated therapeutics from acidic endosomes. Therefore, our goal was to compare the cytotoxic effect of LLO Y406A on cancer cells (RT4) and normal urothelial cells (NPU), and to identify which cell membranes are the primary target of LLO Y406A by viability assays, life-cell imaging, fluorescence, and electron microscopy. LLO Y406A decreased viability, altered cell morphology, provoked membrane blebbing, and induced apoptosis in RT4 cells, while it did not affect NPU cells. LLO Y406A did not cause endosomal escape in RT4 cells, while the plasma membrane of RT4 cells was revealed as the primary target of LLO Y406A. It has been concluded that LLO Y406A has the ability to selectively eliminate cancer urothelial cells through pore-forming activity at the plasma membrane, without cytotoxic effects on normal urothelial cells. This promising selective activity merits further testing as an anti-cancer agent.

## 1. Introduction

Bladder cancer is the fourth most common cancer in men and the eighth most common in women [1,2,3]. While the treatment of muscle-invasive tumors often involves a radical cystectomy, non-muscle-invasive tumors are usually treated locally with cystoscopic tumor excision and anticancer drug post-treatment [2]. The identification of new agents with tumor-specific cytotoxicity for local tumor treatment can decelerate or stop progression and eliminate residual tumor cells post-cystoscopy, resulting in lower healthcare expenses and extended life expectancy. Anti-cancer agents could be small peptides and toxins capable of destroying cancer cells at concentrations that do not significantly affect healthy cells. As a potential therapy for bladder cancer, we investigated the use of the bacterial toxin listeriolysin O (LLO) in urothelial in vitro models. LLO is a pore-forming toxin produced by the facultative intracellular pathogenic bacterium *Listeria monocytogenes* (*L. monocytogenes*) and is its major virulence factor. LLO induces the internalization of *L. monocytogenes* into host cells. This internalization pathway requires the formation of LLO pores in the plasma membrane of the host cells, and subsequent calcium flux controls *L. monocytogenes* internalization [4]. Inside cells, LLO is responsible for the pore formation in endosomal compartments, leading to bacterial escape into the cytosol. Two membrane-active enzymes, a broad-range phospholipase C (PlcB) and a phospahtidylinositol-specific phospholipase C (PlcA) work together with LLO to form pores in membranes of endosomes, called the primary vacuoles [5,6]. LLO belongs to the family of cholesterol-dependent cytolysins that form pores in cholesterol-rich membranes [7,8,9]. Cholesterol concentration above 35% mol, which is typical for membrane rafts, is a prerequisite for efficient binding of LLO to the membranes [10,11,12]. A distinctive property of LLO is its pH-dependent stability, which exhibits an optimum at ~5.5, a condition found in late endosomes [13,14]. While LLO alone is not stable in solution at neutral pH, in the presence of cell membranes, it can rapidly bind to the membrane and form pores even at neutral pH [13,15,16]. To maintain the integrity of the plasma membrane, cells employ many membrane repair mechanisms, i.e., endocytosis, clogging, patching, and vesicle shedding as a defense against the pore-forming activity of cytolysins [17,18]. Membrane repair mechanisms, in particular, are likely to be important determinants of the outcome of *L. monocytogenes* infection.

Many LLO mutants with different pH-dependent pore-forming, membrane-binding, and cholesterol-binding activities have been constructed [16,19,20,21,22]. The LLO mutant with a substitution (Try→Ala) at the site 406 (LLO Y406A) used in this study was shown to be capable to form transmembrane pores at pH 5.7, however, with only minimal pore-forming activity at the neutral pH in vitro [11]. Thus, LLO Y406A can be considered as a favorable membrane-damaging agent for selective action in membranes with an acidic environment.

In the present study, RT4 cells, a model of low-grade tumor derived from papillary superficial human bladder cancer [23], and porcine normal urothelial (NPU) cells were used. NPU cells were accepted as an appropriate surrogate model for healthy human urothelial cells in terms of morphological and functional studies [24,25,26,27]. In previous studies, we showed significantly higher cholesterol and a membrane raft content and a higher activity of endocytosis in RT4 compared to NPU cells [26,28], both of which indicate a specific targeting of LLO Y406A to cancer cells. Herein, we examined cytotoxicity of LLO Y406A on RT4 and NPU cells, its impact on cell morphology, endosomal escape, plasma membrane integrity, and apoptosis. LLO Y406A showed evident cytotoxicity against RT4 cells with the plasma membrane as the primary target for LLO Y406A, while no escape of endosomal contents was observed in these cells. Therefore, we have highlighted LLO Y406A as a selective agent to eliminate cancer urothelial cells, while showing no deleterious effects on normal urothelial cells.

## 2. Results

### 2.1. Cytotoxicity of LLO Y406 and wt LLO on NPU and RT4 Cells

**Figure 1:**
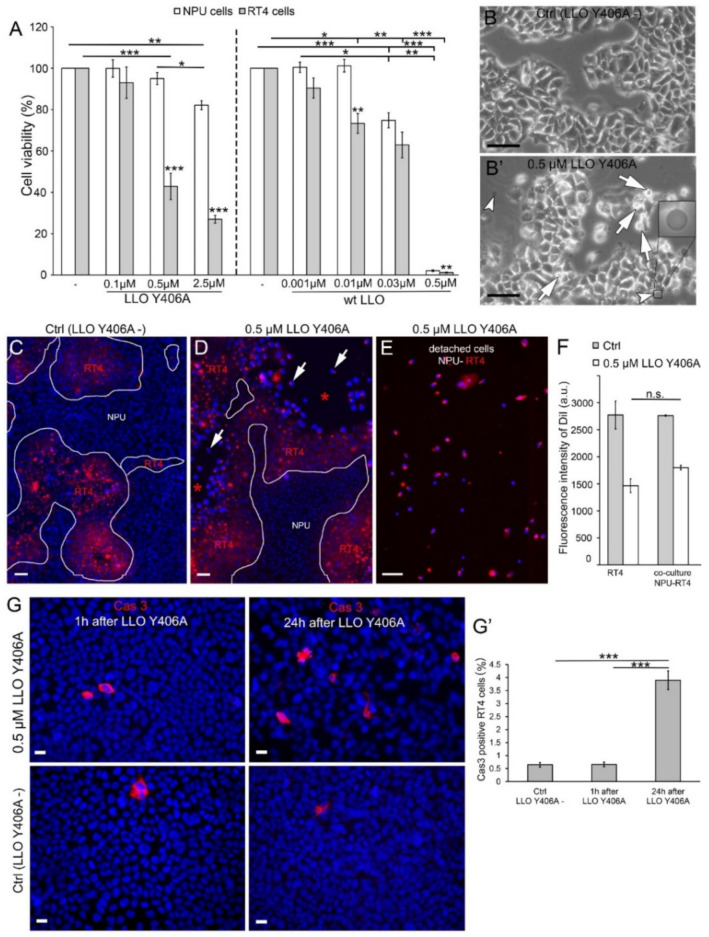
Cell viability and morphology of urothelial cells upon listeriolysin O (LLO) incubation. NPU and RT4 cells were treated for 2 h with LLO Y406A or wt LLO and viabilities measured 1 h post-treatment. (**A**) Cell viability of NPU and RT4 cells in control (−), 0.1, 0.5, and 2.5 µM LLO Y406A, and in 0.001, 0.01, 0.03, and 0.5 µM wt LLO. The data are presented as mean ± SEM, * *p* < 0.05, ** *p* < 0.005, *** *p* < 0.001. (**B**) Morphology of RT4 cells in medium without LLO Y406A and after 0.5 µM LLO Y406A treatment (**B’**). Morphology of RT4 cells changes from polygonal to spherical (arrows). LLO Y406A-treated RT4 cells release vesicles (arrowheads). Cells were imaged with a phase-contrast microscope. The vesicle in the right bottom corner is ×5 enlarged. (**C**) Co-cultures of NPU and RT4 cells, labeled with red lipophilic dye DiI, in control medium. (**D**) Co-cultures after 0.5 µM LLO Y406A treatment have free surfaces (asterisks) with individual RT4 cells (arrows). (**E**) After LLO Y406A treatment, detached cells were collected and imaged. The representative image shows that the majority of cells are RT4 cells. (**F**) Quantification of DiI fluorescence in RT4 monocultures and RT4-NPU co-cultures reveals similar values (*p* > 0.05). (**G**) Immunofluorescence of Cas3 (red) in control untreated RT4 cell and 1 h and 24 h after 0.5 µM LLO Y406A treatment. Nuclei are stained with DAPI (blue). (**G’**) Quantification of the number of apoptotic Cas3-positive RT4 cells, per all RT4 cells in a field of view (fields of view = 10, from three independent experiments). Scale bars: (**B**–**E**) are 50 µm and (**G**) are 20 µm.

We used NPU and RT4 cells to assess cell-dependent susceptibility to LLO Y406A in comparison to the wt LLO. The viability was determined using the CellTiter-Glo assay by measuring cellular ATP levels. Treatment of cells with final 0.1 µM LLO Y406A did not affect the viability of NPU and RT4 cells (Figure 1A). However, treatment with 0.5 µM LLO Y406A significantly decreased the viability of RT4 cells, from 100% to 42.9%, while the same concentration of LLO Y406A resulted in 95.1% viability of NPU cells (Figure 1A). For a comparison with low-grade bladder cancer RT4 cells, we treated high-grade invasive urothelial cancer cells T24 with 0.5 µM LLO Y406A and measured a drop in viability from 100% to 41.1%, which was not significantly different from RT4 cells (Supplementary Figure S1). The viability of both NPU and RT4 cells decreased significantly after incubation with 2.5 µM LLO Y406A compared to untreated NPU and RT4 cells. Both 0.5 µM and 2.5 µM LLO Y406A caused a more prominent decrease in the viability of RT4 cells than NPU cells, however, no significant drop in viability of NPU cells was observed with 0.5 µM LLO Y406A. Application of 0.5 µM wt LLO was detrimental to both cell cultures and resulted in a decrease in viability to 1.2% in RT4 cells and 2.1% in NPU cells. Parallel experiments with wt LLO showed that the viability of NPU cells and RT4 cells decreased at much lower toxin concentrations compared to LLO Y406A. The viability of NPU cells and RT4 cells significantly decreased already with 0.03 µM wt LLO compared to untreated cells (Figure 1A). However, NPU cells remained unaffected by 0.001 and 0.01 µM wt LLO treatment, while RT4 cells were susceptible to 0.01 µM wt LLO and responded with 73.3% viability. After treatment with 0.5 µM LLO Y406A, some RT4 cells changed the morphology from typical polygonal (Figure 1B) to spherical (Figure 1B, arrows) and released extracellular vesicles in the medium (Figure 1B’, arrowheads). To mimic the in vivo situation in two-dimensional culture, we established co-cultures with NPU and RT4 cells (Figure 1C) to reveal the effect of LLO Y406A treatment. In co-cultures, we observed cell-free surfaces surrounded by RT4 cells after 0.5 µM LLO Y406A treatment (Figure 1D, asterisks). The cell-free surfaces resulted from detachment of RT4 cells as confirmed by analysis of the cell suspension in the medium (Figure 1E). The quantification of the fluorescence signal of DiI-labeled RT4 cells in control and LLO Y406A-treated monocultures of RT cells and co-cultures revealed that RT4 cells detached to a similar extent in co-cultures as in monocultures (Figure 1F). These results indicate that NPU cells do not change the sensitivity of RT4 cells to the LLO Y406A.

To compare the short- and long-term effects of LLO Y406A, apoptosis was analyzed 1 and 24 h after 0.5 µM LLO Y406A incubation. Apoptosis in RT4 cells was monitored by immunofluorescence of the active form of caspase 3 (Cas3) (Figure 1G). Incubation with LLO Y406A, induced apoptosis in RT4 cells, which was detected 24 h after incubation with LLO Y406A for 2 h, which corresponds to the maximum time for urine retention in the patient [29]. We quantified the fluorescence signal of Cas3-positive cells (Figure 1G’) and measured a 5.9-fold increase in Cas3 presence when we compared the 24- and 1-h response after LLO Y406A treatment and a 6.0-fold increase in Cas3 when we compared the 24-h response with the untreated RT4 cells.

**Figure 2:**
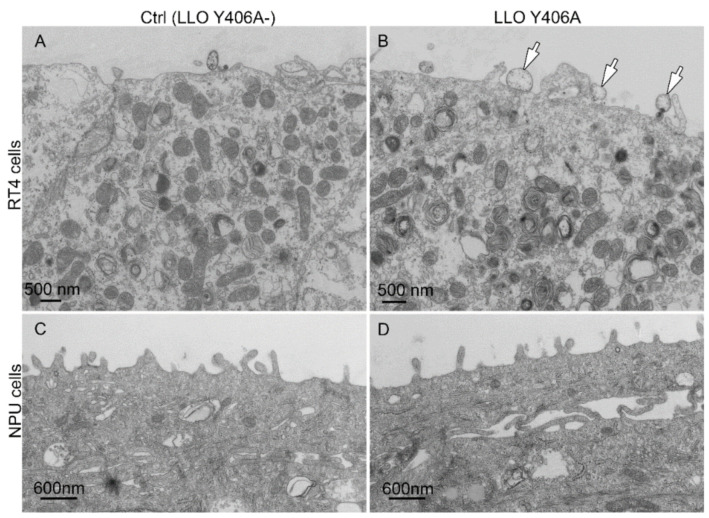
Ultrastructure of urothelial cells after incubation with LLO Y406A. RT4 and normal porcine urothelial (NPU) cells were untreated (**A**,**C**) or treated with 0.5 µM LLO Y406A for 2 h (**B**,**D**). One h after LLO Y406A treatment RT4 cells release extracellular vesicles (arrows, **B**), which are not observed in NPU cells (**D**).

Transmission electron microscopy revealed that in untreated RT4 cells, no vesicles were observed at the cell surface (Figure 2A), while after LLO Y406A treatment vesicles appeared (Figure 2B, arrows). The cell surface of untreated (Figure 2C) and LLO Y406A treated NPU cells (Figure 2D) was similar and vesicles were not observed.

Evaluation of viability after treatments with LLO Y406A and wt LLO revealed LLO Y406A as the preferred toxin variant for selective elimination of >50% RT4 cells while maintaining high viability of NPU cells (~95%). Although 2.5 µM LLO Y406A eliminated RT4 cells even more rigorously, 0.5 µM LLO Y406A outperformed the effect because fewer NPU cells were sacrificed. wt LLO was excluded as a potential agent in cancer cell treatment since concentrations of the wt LLO that were toxic to >40% of RT4 cells were considerably toxic also to NPU cells. Therefore, we chose the final concentration of 0.5 µM LLO Y406A, which was markedly toxic to cancer urothelial cells and non-toxic to normal urothelial cells and used it for the treatment of cancer urothelial cells in the following experiments.

### 2.2. LLO Y406A Does Not Cause Endosomal Escape and Is Mainly Located at He Plasma Membrane

**Figure 3:**
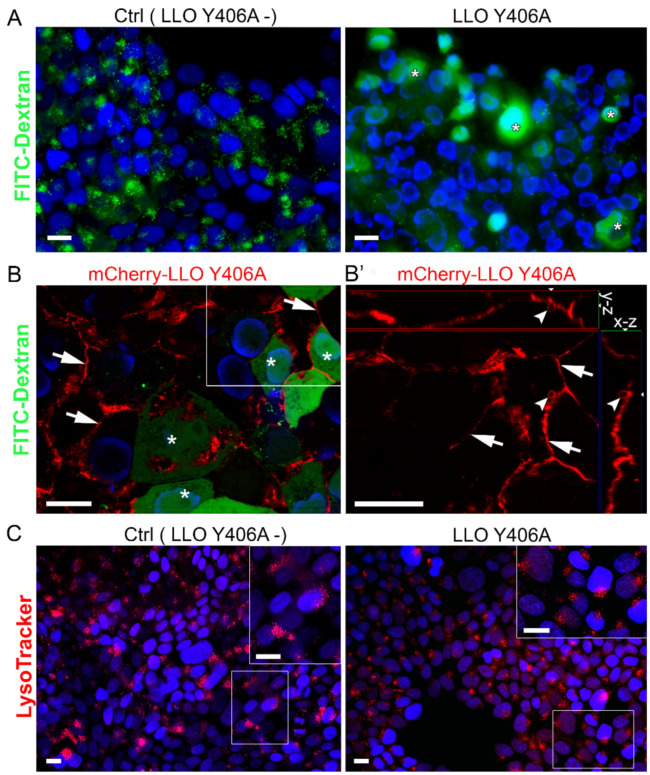
LLO Y406A does not cause endosomal escape, but plasma membrane perforation and blebbing in RT4 cells. (**A**) FITC-dextran shows dotted pattern fluorescence in control (LLO Y406A-) cells. After co-incubation with 0.5 µM LLO Y406A and FITC-dextran, part of RT4 cells exhibits the cytosolic distribution of dextran (asterisks). (**B**) FITC-dextran and 5.0 µM mCherry-LLO Y406A co-incubation results in the plasma membrane labeling with mCherry-LLO Y406A (arrows). Part of the cells shows a homogenous distribution of dextran (asterisks). (**B’**) Magnification of the region in B (white frame) is shown in the x-z and y-z view. Plasma membrane (arrows) and extracellular vesicles (arrowheads), emanating from the plasma membrane, are labeled with mCherry-LLO Y406A. (**C**) The distribution of LysoTracker is similar in untreated and in LLO Y406A-treated cells and has a dotted pattern shown in the magnified insets. Nuclei are stained with DAPI (blue). Scale bars are 20 µm.

Fluorescent dextrans are commonly used as macropinocytotic probes to study endocytosis. We co-incubated LLO Y406A and FITC-dextran to identify dispersion of endosomal content as an indication of the pore-forming activity of LLO Y406A in endosomal membranes. The pattern of FITC-dextran distribution was dotted in the control untreated RT4 cells (Figure 3A), reflecting endosomes with intact membranes. One h after treatment with 0.5 µM LLO Y406A, a portion of RT4 cells displayed homogeneous FITC-dextran distribution (Figure 3A, asterisks). Incubation with 5.0 µM mCherry-LLO Y406A and dextran revealed preferential labeling at the plasma membrane, indicating binding of mCherry-LLO Y406A to the cell surface (Figure 3B,B’, arrows). We treated RT4 cells for 2 h with 5.0 µM mCherry-LLO Y406A, which decreased the viability of RT4 cells to 81.9% (Supplementary Figure S2). mCherry-LLO Y406A binding to the plasma membrane presumably led to the pore-formation that enabled leaking of dextran into cells and its homogenous distribution in RT4 cells. Furthermore, cells with mCherry-LLO Y406A labeling released vesicles (Figure 3B’, arrowheads), presumably as an outcome of the repair mechanism [17]. Utilizing another marker for acidic endosomal compartments LysoTracker, we found a similar dotted staining pattern in control and LLO Y406A-treated cells (Figure 3C), indicating that LLO Y406A had no activity in endosomes.

At the fluorescence-microscopy level, neither dextran nor LysoTracker revealed dispersion of endosomal content into the cytosol after LLO Y406A treatment. Plasma membrane labeling with mCherry-LLO Y406A implies that LLO Y406A perforates the plasma membrane of RT4 cells, thereby allowing passage of dextran into the cytosol.

**Figure 4:**
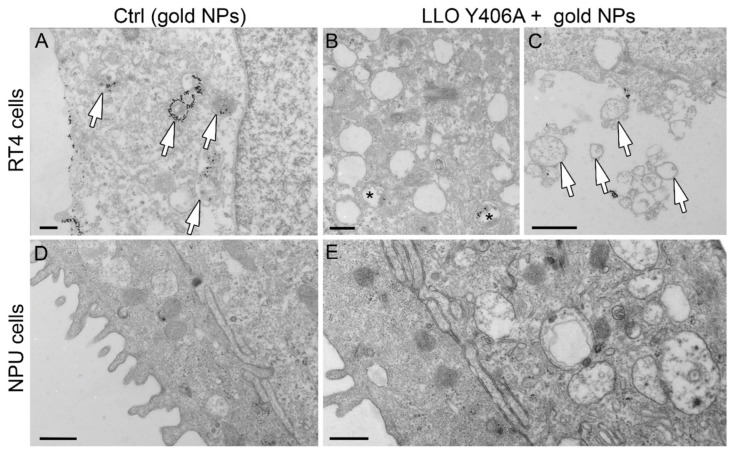
LLO Y406A does not cause endosomal escape of gold nanoparticles (NPs) but causes blebbing in RT4 cells. RT4 cells internalize gold NPs after 2-h incubation with gold NPs followed by 1-h incubation in the control medium. (**A**) Gold NPs are concentrated inside endosomes (arrows). (**B**) After co-treatment with 0.5 µM LLO Y406A and gold NPs, gold NPs persist in endosomes (asterisks) and show no release of gold NPs into the cytosol. (**C**) LLO Y406A treatment causes the release of extracellular vesicles from the cell surface (arrows). (**D**) NPU cells do not internalize gold NPs. (**E**) After co-treatment with LLO Y406A and gold NPs, no gold NPs are released in the cytosol of NPU cells (**E**). Scale bars (**A**–**C**) are 300 nm, (**D**,**E**) are 600 nm.

To reveal the escape of endosomal content after LLO Y406A treatment, which could not be resolved by light microscopy, we utilized transmission electron microscopy. As a proof of concept, we used gold nanoparticles (gold NPs) as candidate markers to be co-delivered with LLO Y406A. We prepared gold NPs with an average size of 10 ± 2 nm [30] (Supplementary Figure S3). In the control medium, RT4 cells internalized gold NPs (Figure 4A, arrows) by endocytosis as is evident from gold NPs presence in endosomes (Figure 4A, arrows). In RT4 cells, which were treated with gold NPs and 0.5 µM LLO Y406A, gold NPs persisted inside endosomes (Figure 4B, asterisk) and no NPs were distributed freely within the cytoplasm. In RT4 cells treated with LLO Y406A, extracellular vesicles were frequently observed (Figure 4C, arrows). On the other hand, in NPU cells that were treated only with gold NPs, no internalization of NPs was observed (Figure 4D) as was previously discovered for magnetic NPs [28]. Furthermore, LLO Y406A and gold NPs co-incubation did not cause any blebbing of the plasma membranes in NPU cells (Figure 4E).

Our results confirmed that LLO Y406A did not perforate endosomal membranes, because gold NPs were not released from endosomes into the cytosol. We speculate that the prevention of endosomal escape could be due to the absence of necessary virulence factors, membrane-active phospholipase C enzymes that contribute to pore formation in endosomes during *Listeria* infection [31] or by the increased physical resistance of the endolysosomal compartment in these cells [9]. However, our results strongly demonstrate that LLO Y406A is mainly attached to the plasma membrane and acts there and hence the amount of internalized LLO Y406A is probably too low for pore formation in endosomes.

### 2.3. LLO Y406A Induces Calcium Influx and Cell Blebbing in RT4 Cells

**Figure 5:**
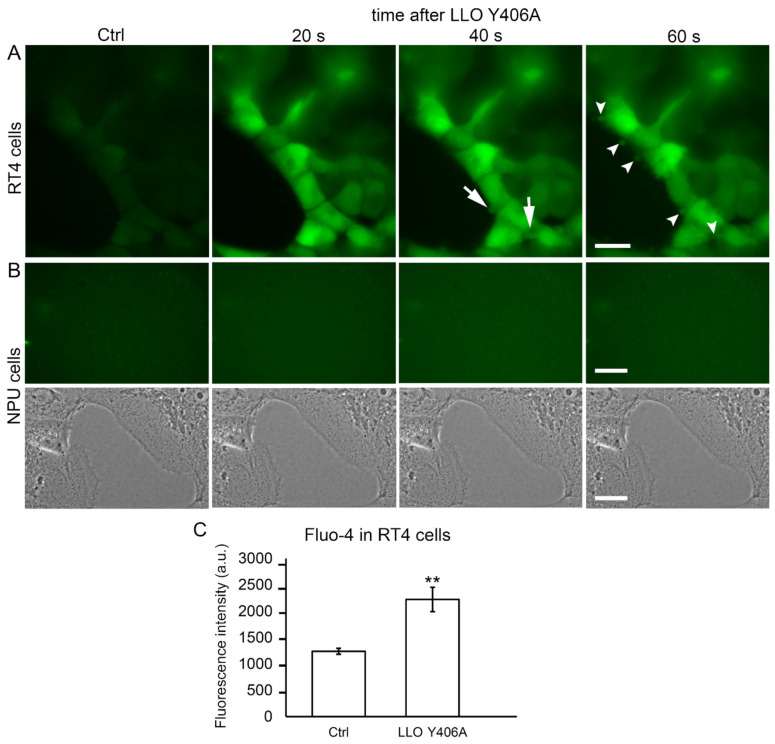
LLO Y406A induces an influx of calcium into RT4 cells. (**A**) Fluorescence of the calcium indicator Fluo-4 in RT4 cells without LLO Y406A and 20, 40, and 60 s after 0.5 µM LLO Y406A incubation. Cell blebbing occurs 40 s after LLO Y406A incubation (arrows), followed by the release of extracellular vesicles 60 s after LLO Y406A incubation (arrowheads). (**B**) Fluorescence of Fluo-4 in NPU cells without LLO Y406A and 20, 40, and 60 s after 0.5 µM LLO Y406A incubation. LLO Y406A did not induce calcium influx in NPU cells. No fluorescence of Fluo-4 (upper row) and no changes in morphology (bottom row) appeared during 60 s after 0.5 µM LLO Y406A incubation. Images of RT4 and NPU cells were acquired with the same exposure time (558.47 ms). Scale bars are 20 µm. (**C**) Quantification of Fluo-4 fluorescence, presented as gray values of the fluorescence intensity signal (a.u.), in RT4 cells in the absence of LLO Y406A and 60 s after treatment with LLO Y406A. Presented are averages ± SEM, ** *p* < 0.005.

It has been shown before that the pore-forming activity of LLO is not necessarily restricted to intracellular targets but can also be directed to the plasma membrane of cells [8,15,32,33,34]. Thus, we utilized Fluo-4, a calcium indicator, to examine the activity of LLO Y406A at the plasma membrane of RT4 cells. An evident increase in fluorescence of Fluo-4 inside RT4 cells was found already 20 s after LLO Y406 incubation in comparison to untreated cells (Figure 5A). This calcium influx was immediately followed by blebbing, i.e., 40 s after LLO Y406A treatment (Figure 5A, arrows). Blebbing progressed to the release of extracellular vesicles into the extracellular space 60 s after LLO Y406A application (Figure 5A, arrowheads). By the same experimental design with NPU cells, no Fluo-4 fluorescence or blebbing was observed (Figure 5B). In RT4 cells, the significant increase in intercellular calcium after LLO Y406A application was confirmed by quantifying the gray values of the Fluo-4 fluorescence intensity. The latter was shown to be 1.8-fold higher in LLO Y406A-treated than in untreated RT4 cells (Figure 5C).

This examination revealed that LLO Y406A activity is targeted specifically to the plasma membrane of RT4 cells, resulting in calcium influx, immediate membrane blebbing, and shedding of extracellular vesicles.

## 3. Discussion

The novelty of this study is the selective pore-forming activity of LLO Y406A for urothelial cells in vitro where cancer cells respond with high sensitivity, while the sensitivity of normal cells is only negligible. To our knowledge, this is the first study in which wt LLO or its mutants have been used on urothelial cells. To date, wt LLO activity has been studied in a variety of other cell types, including colon epithelial cells Caco-2 and HT-29/B6, embryonic kidney cells HEK 293, human brain endothelial cells, and Jurkat cells, each without comparing the response of wt LLO in cancer vs. normal cells [34,35,36,37,38]. Herein, we initially planned to use LLO Y406A, designed for activity at a narrow pH optimum of 5–6 [11] to prevent pore-forming activity at the plasma membrane with usually neutral pH surrounding, which should induce selective pore-forming activity in acidic endosomes. In RT4 cells, 0.5 µM LLO Y406A treatment decreased viability to 43%, whereas normal urothelial cells survived the same treatment with 95%. A larger proportion of RT4 cells died presumably due to membrane damage and detachment from the growth surface caused by LLO Y406A activity and partially by the apoptosis. This effect was detected 24 h after LLO Y406A treatment and supports a remarkable cytotoxic activity of LLO Y406A. These results highlighted the mutant LLO Y406A as a promising therapeutic agent due to (a) high selectivity to attack RT4 cells and (b) high efficiency to eliminate RT4 cells in general and in comparison to wt LLO. The low toxicity of LLO Y406A to NPU cells is an important advantage of this agent and suggests its potential for in vivo studies, where NPU cells tend to be protected from impairment in terms of maintaining the blood-urine barrier and minimizing side effects. Co-cultures of RT4 and NPU cells proved LLO Y406A selectivity for RT4 cells. A similar trend of selective elimination of RT4, but not NPU cells, was observed after incubation with the cholesterol-sphingomyelin binding protein ostreolysin A/pleurotolysin B (OlyA/PlyB) [26]. Since OlyA/PlyB and LLO Y406A require cholesterol for binding, the difference in activity on RT4 and NPU cells is probably connected to the higher concentration of cholesterol in RT4 cells (23 µg/mg proteins) compared to NPU cells with 17 µg cholesterol per mg proteins [26].

In addition to cholesterol content, another possible reason for the cell-type specific action of LLO Y406A is the acidic microenvironment that likely surrounds RT4 cells. The acidity is provided by lactate as a metabolic product of cancer cell-specific glycolysis known as the Warburg effect [39,40]. This effect is strongly implicated in bladder cancer as it is associated with an aggressive tumor and promotes metastatic spread by exerting an inhibitory action on anti-cancer immune effectors [41,42,43]. Even though RT4 cells were incubated with LLO Y406A in a pH-neutral culture medium, it is likely, that the local acidity at the plasma membrane was still suitable for the activity of this cytolysin. The mCherry-LLO Y406A labeling at the plasma membrane of RT4 cells provided evidence in this direction. LLO Y406A tropism to RT4 urothelial cells might be favored by a specific class of glycans that are absent from the NPU cells [44,45]. Further studies are needed to determine whether RT4 cells are decorated with the glycan class that represents LLO receptors [46].

In addition to the evident binding of LLO Y406A to the plasma membrane, its pore-forming activity has been proven with the calcium influx into RT4 cells. It happened within the first 20 s after LLO Y406A application. However, the exact size of pores has yet to be determined. According to the hydrodynamic diameter of 2.3 nm of 3 kDa dextran that entered the cytosol and ~10 nm of gold NPs that could not enter, we can roughly estimate the pore size to be between 2 and 10 nm [47]. We found that RT4 cells responded rapidly to LLO Y406A with blebbing and the release of extracellular vesicles. It has been shown that blebbing is a repair mechanism by which pores smaller than 100 nm become quarantined and detached by extracellular vesicles [18,48,49]. Streptolysin O, a cholesterol-dependent cytolysin, caused sequestration of the damaged membrane around the pores and subsequent shedding into extracellular vesicles in Teu-2 urothelial cells and 3T3 fibroblasts [50,51]. Thus, we can speculate that membrane blebbing is a general mechanism in urothelial cells in response to the pore-forming toxins. The mechanism of blebbing probably helps to recover the integrity of the plasma membrane and contributes to cell recovery and proliferation. On the other hand, released LLO Y406A vesicles (microvesicles) can target other RT4 cells and continue with the pore-forming activity, thereby prolonging and enhancing the anti-cancer effect of LLO Y406A. For example, biologically active LLO-containing vesicles, toxic to human breast cancer cells and murine macrophages, were formed by epithelial cells after infection with *L. monocytogenes* [52].

## 4. Materials and Methods

### 4.1. Cell Cultures

Monocultures: Human cancer urothelial RT4 and T24 cells (ATTC, Manassas, VA, USA) were cultured in advanced Dulbecco’s modified Eagle’s medium (A-DMEM)/F12 (1:1), 5% heat-inactivated fetal bovine serum (FBS), 10,000 U/mL penicillin, and 10,000 µg/mL streptomycin solution (determined as control medium) at 37 °C in a humidified atmosphere with 5% CO_2_. The porcine normal urothelial (NPU) cells from the fourth to twelfth passage were prepared from at least three porcine normal urinary bladders (biological replicates) as described previously [53]. NPU cells were cultured in MCDB153 (Sigma-Aldrich, Taufkirchen, Germany)/A-DMEM (1:1), 2.5% FBS, 0.1 mM phosphoethanolamine (Sigma), 0.5 μg/mL hydrocortisone (Sigma), 5 μg/mL insulin (Sigma), 4 mM glutamax, 10,000 U/mL penicillin, and 10,000 µg/mL streptomycin solution (control medium). The culture media and supplements were purchased from Gibco, Invitrogen (Vienna, Austria), unless otherwise stated.

Co-cultures: The NPU cells (1 × 10^5^ cells/cm^2^) and RT4 cells (1 × 10^5^ cells/cm^2^) were cultured for 2 days in Petri dishes or in 96-well plates. To discriminate RT4 cells from NPU cells, RT4 cells were labeled with the orange—red-fluorescent lipophilic dye DiI (1,1′-Dioctadecyl-3,3,3′,3′-Tetramethylindocarbocyanine Perchlorate; V22885, Thermo Fisher Scientific, Waltham, MA, USA) before co-culturing. Briefly, 4 µl DiI was added to 1 × 10^6^ RT4 cells for 30 min at 37 °C, cells were rinsed five times with fresh control medium and seeded with unlabeled NPU cells. Co-cultures were left untreated or were treated with 0.5 µM LLO Y406A, grown for an hour in a control medium. The medium was collected and detached cells were pelleted (200× *g*, 5 min) and fixed in 4% formaldehyde in phosphate-buffered saline (PBS; pH 7.4). The attached cells were either fixed in 4% formaldehyde in phosphate-buffered saline (PBS; pH 7.4) or DiI fluorescence was measured with a microplate reader (Safire2, Tecan, Mannedorf, Switzerland). Fixed cells were rinsed with PBS and embedded in Vectashield with DAPI (Vector Laboratories, Burlingamme, CA, USA), and analyzed with 10×/NA 0.3 objective on a fluorescence microscope AxioImager Z.1. (Zeiss, Oberkochen, Germany).

### 4.2. Cell Viability Measurement

The RT4 cells and NPU cells were cultured for 2 days in 96-well plates (Costar, Kennebunk, ME, USA), each at a seeding density of 1 × 10^5^ cells/cm^2^. Cells were treated with 0.1, 0.5, 2.5 µM LLO Y406A and 0.005, 0.01, 0.03 µM wild-type LLO (wt LLO) for 2 h in control medium, washed and left in fresh control medium for 1 h, or were untreated. Cell viability was tested with CellTiter-Glo Luminescent Cell Viability Assay (Promega, Madison, WI, USA) according to the manufacturer’s instructions. The luminescence was measured using a microplate reader (Safire2). Data from viability assays are expressed as percentages of luminescence of treated to untreated cells, as an average ± standard error of the mean (SEM) of 3 independent experiments, each performed in triplicate.

### 4.3. Preparation, Expression and Purification of Recombinant Proteins

The wt LLO and LLO Y406A were prepared as previously described [11]. Proteins were expressed in *Escherichia coli* BL21(DE3) pLysS cells in Terrific Broth medium. Cells were grown at 37 °C with shaking until A_600_ reached ~1 and protein expression was induced with 0.5  mM isopropyl 1-thio-β-D-galactopyranoside. After shaking for 20  h at 20  °C, the cells were harvested by centrifugation at 4  °C and 4410× *g* for 5 min. Cells were resuspended in a minimal amount of lysis buffer (50  mM NaH_2_PO_4_/Na_2_HPO_4_, 250  mM NaCl, 10% (*v*/*v*) glycerol, pH 6.5) and 2-sulfanylethanol was added to a final concentration of 5  mM. Protease inhibitor phenylmethanesulfonyl fluoride was added to a final concentration of 2  mM. The cell suspension was sonicated and debris was removed by centrifugation at 50,000× *g*, 4 °C for 1 h. The supernatant was filtered through 0.22  μm filter, aliquoted, and frozen at −80  °C until use. For protein purification, the cell lysate was loaded to Ni-NTA, (Qiagen, Hilden, Germany) column, washed extensively with the wash buffer (50  mM NaH_2_PO_4_/Na_2_HPO_4_, 250  mM NaCl, 60  mM imidazole, pH 6.5), and the bound fraction eluted with the same wash buffer except the imidazole concentration was 300  mM. Tobacco etch protease (TEV) was added to a final concentration of ~30  μg/mL and the solution was dialyzed 1:60 overnight at 4 °C in a dialysis buffer (30  mM NaH_2_PO_4_/Na_2_HPO_4_, 150  mM NaCl, pH 6.5). The dialyzed sample was loaded again on Ni-NTA column and the unbound fraction, containing purified protein was concentrated with Amicon Ultra 30  kDa MWCO (Merck, Darmstadt, Germany) for the final step, size exclusion chromatography. The Superdex 200 column (GE Healthcare, Chicago, IL, USA) was equilibrated with GF buffer (20  mM 2-(N-morpholino)ethanesulfonic acid (MES), 150  mM NaCl, pH 5.7). Fractions of pure protein (determined with SDS-PAGE) were pooled, concentrated, aliquoted, and flash frozen in liquid nitrogen and stored at −80  °C.

The gene for mCherry-LLO Y406A recombinant protein was inserted into pET17b plasmid. The mCherry was linked to LLO Y406A at the N-terminus using a linker with amino acid sequence GGGSGGGSPR. C-terminally to the LLO Y406A sequence, the TEV protease cleavage site, and a hexa-histidine tag were added. mCherry-LLO Y406A protein was expressed in *E. coli* BL21(DE3) cells as described above for wt LLO and LLO Y406A. The cell lysate was loaded onto Ni-NTA (Qiagen, Hilden, Germany) column for protein purification. The column was washed with 50 mM NaH_2_PO_4_/Na_2_HPO_4_ pH 7.5, 250 mM NaCl, 5% *v*/*v* glycerol and the bound recombinant protein was eluted with 50 mM NaH_2_PO_4_/Na_2_HPO_4_ pH 7.5, 250 mM NaCl, 300 mM NaCl, 5% *v*/*v* glycerol. 6xHis-Tag was cleaved with TEV protease in dialysis buffer (30 mM NaH_2_PO_4_/Na_2_HPO_4_ pH 7.5, 150 mM NaCl, 5% glycerol) overnight at 4 °C. After dialysis, the protein solution was loaded to the Ni-NTA column and the unbound fraction with the purified protein was concentrated in Amicon Ultra 30 kDa MWCO (Merck, Darmstadt, Germany) and the buffer exchanged so the final protein solution was in 20 mM NaH_2_PO_4_/Na_2_HPO_4_ pH 7.5, 150 mM NaCl, 5% glycerol, 1 mM DTT) and stored at −80 °C until used.

Stock solutions of LLO-Y406A (69.1 µM), mCherry-LLO Y406A (22.8 µM) and wt LLO (101.0 µM) were mixed in control medium alone or combination with FITC-dextran or gold nanoparticles (see preparation below) at 22 °C in a laminar flow hood. 

### 4.4. Endosomal Escape

To reveal endosomal escape, RT4 cells were co-incubated with 0.5 µM LLO Y406A and 0.5 mg/mL FITC-dextran (3kDa, D3306, Thermo Fisher Scientific, Waltham, MA, USA) for 2 h, washed in control medium, and cultured in control medium for 1 h. Alternatively, RT4 cells were co-incubated with 5.0 µM mCherry-LLO Y406A and FITC-dextran following the same protocol. Then cells were then fixed in 4% formaldehyde in PBS (pH 7.4) at 22 °C for 15 min and mounted in Vectashield with DAPI (4,6-diamidino-2-phenylindole) for nuclear staining (Vector Laboratories). Additionally, LysoTracker Red DND-99 (L7528, Molecular Probes Life Technologies, Waltham, OR USA) was used. In brief, RT4 cells were incubated with 50 nM LysoTracker at 37 °C for 45 min. Cells were then washed and LLO Y406A was added to a final concentration of 0.5 µM for 2 h and washed with control medium. One h later, cells were fixed in 4% formaldehyde in PBS at 22 °C for 15 min and mounted in Vectashield with DAPI. Cells were analyzed using a 20×/NA 0.75 and 63×/NA 1.4 objectives on a fluorescence microscope AxioImager Z.1 with an Apotome device for optical section generation (Zeiss).

### 4.5. Immunolabeling

For caspase 3 (Cas3) labeling, RT4 cells were incubated with 0.5 µM LLO Y406A for 2 h, washed in control medium, and cultured for 1 and 24 h in control medium, then fixed in 4% formaldehyde at 22 °C for 15 min, and incubated in a blocking-permeabilization buffer (0.5% bovine serum albumin, 0.1% saponin, 0.1% gelatin, 50 mM NH_4_Cl, 0.02% NaN_3_) at 37 °C for 30 min. Then cells were incubated with rabbit anti-cleaved Cas3 antibodies (1:100, ab2302, Abcam, Cambridge, UK) overnight at 4 °C, washed with PBS, and incubated with Alexa Fluor 555-conjugated anti-rabbit secondary antibodies (1:400, Thermo Fisher Scientific, Waltham, MA, USA) at 37 °C for 30 min. In parallel, untreated RT4 cells were processed for Cas3 immunolabeling using the same protocol as for LLO Y406A-treated cells. The cells were mounted in Vectashield with DAPI for nuclear staining and observed under fluorescence microscope AxioImager Z1. Cas3 positive cells and nuclei were counted using the Cell Counter Plugin (Fiji, release 2.2.2., NIH, Bethesda, MD, USA).

### 4.6. Fluo-4 AM Calcium Staining

The RT4 and NPU cells (1 × 10^5^ cells/cm^2^) were seeded on glass-bottom dishes (MatTekCorporation, Ashland, MA, USA) and grown for 2 days. Cells were washed with HBSS+ (Hanks buffer with Ca^2+^, Mg^2+^, without Phenol Red, 10 mM HEPES, 10 mM glucose) buffer and incubated with 2 μM Fluo-4 AM (*F14201*, Molecular Probes Life Technologies, Eugene, OR, USA) at 37 °C for 45 min in the dark. Cells were then washed with HBSS+ buffer and FluoroBright DMEM microscopy medium without FBS (Gibco) was added to the cells. Imaging was made with Zeiss Axio Imager Z.1 fluorescence microscope at 37 °C (Filter set 10, 488010, Zeiss) and images were acquired using the AxioVision program. Then LLO Y406A was added at the final concentration 0.5 µM and 20 s later images were taken at 10-s intervals with the same settings as in pre-treatment. Gray values of the fluorescence intensity (a.u.) were measured with AxioVision 4.8 software (Zeiss) in three independent experiments, each time in 10 cells.

### 4.7. Generation of Gold Nanoparticles

The procedure for gold nanoparticles (gold NPs) preparation was adopted from Hayat (Hayat 1989). Gold NPs were prepared by reduction of HAuCl_4_ with sodium citrate. The starting solution of 106 mL of 2.2 mM (0.064%, *w*/*v*) sodium citrate was brought to a boil and 1 mL of 24.3 mM HAuCl_4_ (0.955%, *w*/*v*) was added with rapid mixing. The reaction was completed within 1–2 min and the solution was further boiled for 15 min. Gold NPs in solution were centrifuged at 6500× *g* for 30 min at 4 °C. Nine tenths of the supernatant was discarded to increase the concentration of gold NPs in the solution. Next, 5 μL of the gold NPs solution were deposited onto copper grids and air-dried. Images of gold NPs were taken at 20,000–100,000× magnifications with a Philips CM100 transmission electron microscope (Philips, Tokyo, Japan) at 80 kV using an AMT camera (Advanced Microscopy Techniques Corp., Woburn, MA, USA).

### 4.8. Transmission Electron Microscopy

The RT4 and NPU cells treated with 0.5 µM LLO Y406A, with 0.5 µM LLO Y406A and gold NPs, and the untreated cells were fixed with 4% formaldehyde and 2% glutaraldehyde in 0.1 M cacodylate buffer for 2 h at 4 °C. After overnight washing in 0.33 M sucrose in cacodylate buffer, the cells were incubated with 1% OsO_4_ for 1 h at 22 °C. The cells were then dehydrated and embedded in Epon (Serva, Heidelberg, Germany). Ultrathin sections were stained with uranyl acetate and lead citrate (Merck, Darmstadt, Germany). The sections were examined with a Philips CM100 transmission electron microscope at 80 kV using an AMT camera.

### 4.9. Statistical Analysis

The data are presented as average ± SEM of three independent experiments, each performed in triplicates. Data were analyzed with Student’s two-tailed t-test or one-way Anova test (MS Excel for Windows 2011, version 14.3.2). Statistically significant results were represented by * *p* < 0.05; ** *p* < 0.005; and *** *p* < 0.001.

## 5. Conclusions

LLO Y406A has great selectivity for the urothelial cancer cells, which is the most important feature of an effective anticancer drug. We envision the use of LLO Y406A in combination with other conventional drugs such as Bacillus Calmette–Guérin (BCG) or mitomycin C that are used after bladder tumor resection to destroy remnant tumor cells [54,55]. LLO Y406A fulfils many attributes as an anti-cancer agent: it is a small 56 kDa protein, soluble in urine, with highly selective activity at the plasma membrane of cancer urothelial cells that results in the efficient elimination of cancer urothelial cells. By intravesical instillation, LLO Y406A could be directly applied, without any preparation of the drug, i.e., encapsulation into liposomes. Many advantages of this agent merit further investigation of LLO Y406A as a specific therapeutic against bladder papillary tumors which we intend to examine in animal models of bladder cancer such as orthotopic bladder cancer models to approach the translation of this experimental work to the clinics.

## Data Availability

The data presented in this study are available on request from the corresponding author.

## Supplementary Materials

The following are available online at https://www.mdpi.com/1422-0067/22/7/3305/s1, Figure S1: Comparison in the viability of T24 and RT4 cells after 0.5 µM LLO Y406A treatment, Figure S2: The viability of RT4 cells after mCherry-LLO Y406A treatment, Figure S3: Transmission electron microscopy of gold NPs.

## References

[1] Bray F., Ferlay J., Soerjomataram I., Siegel R.L., Torre L.A., Jemal A. (2018). Global cancer statistics 2018: GLOBOCAN estimates of incidence and mortality worldwide for 36 cancers in 185 countries. CA Cancer J. Clin..

[2] Richters A., Aben K.K.H., Kiemeney L. (2020). The global burden of urinary bladder cancer: An update. World J. Urol..

[3] Kirkali Z. (2006). The future of oncological urology in Europe: Are we prepared?. Eur. Urol..

[4] Vadia S., Seveau S. (2014). Fluxes of Ca^2+^ and K^+^ are required for the listeriolysin O-dependent internalization pathway of *Listeria monocytogenes*. Infect. Immun..

[5] Mostowy S., Cossart P. (2012). Virulence factors that modulate the cell biology of *Listeria* infection and the host response. Adv. Immunol..

[6] Alberti-Segui C., Goeden K.R., Higgins D.E. (2007). Differential function of *Listeria monocytogenes* listeriolysin O and phospholipases C in vacuolar dissolution following cell-to-cell spread. Cell. Microbiol..

[7] Coconnier M.H., Lorrot M., Barbat A., Laboisse C., Servin A.L. (2000). Listeriolysin O-induced stimulation of mucin exocytosis in polarized intestinal mucin-secreting cells: Evidence for toxin recognition of membrane-associated lipids and subsequent toxin internalization through caveolae. Cell. Microbiol..

[8] Hamon M.A., Ribet D., Stavru F., Cossart P. (2012). Listeriolysin O: The Swiss army knife of *Listeria*. Trends Microbiol..

[9] Seveau S. (2014). Multifaceted activity of listeriolysin O, the cholesterol-dependent cytolysin of *Listeria monocytogenes*. Subcell. Biochem..

[10] Bavdek A., Gekara N.O., Priselac D., Gutierrez Aguirre I., Darji A., Chakraborty T., Macek P., Lakey J.H., Weiss S., Anderluh G. (2007). Sterol and pH interdependence in the binding, oligomerization, and pore formation of Listeriolysin O. Biochemistry.

[11] Kisovec M., Rezelj S., Knap P., Cajnko M.M., Caserman S., Flašker A., Žnidaršič N., Repič M., Mavri J., Ruan Y. (2017). Engineering a pH responsive pore forming protein. Sci. Rep..

[12] Schuerch D.W., Wilson-Kubalek E.M., Tweten R.K. (2005). Molecular basis of listeriolysin O pH dependence. Proc. Natl. Acad. Sci. USA.

[13] Bavdek A., Kostanjsek R., Antonini V., Lakey J.H., Dalla Serra M., Gilbert R.J., Anderluh G. (2012). pH dependence of listeriolysin O aggregation and pore-forming ability. FEBS J..

[14] Schnupf P., Portnoy D.A. (2007). Listeriolysin O: A phagosome-specific lysin. Microbes Infect..

[15] Vadia S., Arnett E., Haghighat A.C., Wilson-Kubalek E.M., Tweten R.K., Seveau S. (2011). The pore-forming toxin listeriolysin O mediates a novel entry pathway of *L. monocytogenes* into human hepatocytes. PLoS Pathog..

[16] Podobnik M., Marchioretto M., Zanetti M., Bavdek A., Kisovec M., Cajnko M.M., Lunelli L., Dalla Serra M., Anderluh G. (2015). Plasticity of listeriolysin O pores and its regulation by pH and unique histidine [corrected]. Sci. Rep..

[17] Brito C., Cabanes D., Sarmento Mesquita F., Sousa S. (2019). Mechanisms protecting host cells against bacterial pore-forming toxins. Cell. Mol. Life Sci..

[18] Etxaniz A., Gonzalez-Bullon D., Martin C., Ostolaza H. (2018). Membrane Repair Mechanisms against Permeabilization by Pore-Forming Toxins. Toxins.

[19] Nomura T., Kawamura I., Kohda C., Baba H., Ito Y., Kimoto T., Watanabe I., Mitsuyama M. (2007). Irreversible loss of membrane-binding activity of *Listeria*-derived cytolysins in non-acidic conditions: A distinct difference from allied cytolysins produced by other Gram-positive bacteria. Microbiology.

[20] Plaza-Ga I., Manzaneda-González V., Kisovec M., Almendro-Vedia V., Muñoz-Úbeda M., Anderluh G., Guerrero-Martínez A., Natale P., López Montero I. (2019). pH-triggered endosomal escape of pore-forming Listeriolysin O toxin-coated gold nanoparticles. J. Nanobiotechnol..

[21] Watanabe I., Nomura T., Tominaga T., Yamamoto K., Kohda C., Kawamura I., Mitsuyama M. (2006). Dependence of the lethal effect of pore-forming haemolysins of Gram-positive bacteria on cytolytic activity. J. Med. Microbiol..

[22] Malet J.K., Cossart P., Ribet D. (2017). Alteration of epithelial cell lysosomal integrity induced by bacterial cholesterol-dependent cytolysins. Cell. Microbiol..

[23] Sanchez-Carbayo M., Socci N.D., Charytonowicz E., Lu M., Prystowsky M., Childs G., Cordon-Cardo C. (2002). Molecular profiling of bladder cancer using cDNA microarrays: Defining histogenesis and biological phenotypes. Cancer Res..

[24] Kreft M.E., Di Giandomenico D., Beznoussenko G.V., Resnik N., Mironov A.A., Jezernik K. (2010). Golgi apparatus fragmentation as a mechanism responsible for uniform delivery of uroplakins to the apical plasma membrane of uroepithelial cells. Biol. Cell.

[25] Resnik N., Prezelj T., De Luca G.M.R., Manders E., Polishchuk R., Veranič P., Kreft M.E. (2018). Helical organization of microtubules occurs in a minority of tunneling membrane nanotubes in normal and cancer urothelial cells. Sci. Rep..

[26] Resnik N., Repnik U., Kreft M.E., Sepčić K., Maček P., Turk B., Veranič P. (2015). Highly Selective Anti-Cancer Activity of Cholesterol-Interacting Agents Methyl-β-Cyclodextrin and Ostreolysin A/Pleurotolysin B Protein Complex on Urothelial Cancer Cells. PLoS ONE.

[27] Višnjar T., Kreft M.E. (2015). The complete functional recovery of chitosan-treated biomimetic hyperplastic and normoplastic urothelial models. Histochem. Cell Biol..

[28] Lojk J., Bregar V.B., Strojan K., Hudoklin S., Veranič P., Pavlin M., Kreft M.E. (2018). Increased endocytosis of magnetic nanoparticles into cancerous urothelial cells versus normal urothelial cells. Histochem. Cell Biol..

[29] Kim S.H., Kim S.R., Yoon H.Y., Chang I.H., Whang Y.M., Cho M.J., Kim M.J., Kim S.Y., Lee S.J., Choi Y.W. (2017). Poloxamer 407 Hydrogels for Intravesical Instillation to Mouse Bladder: Gel-Forming Capacity and Retention Performance. Korean J. Urol. Oncol..

[30] Hayat M.A. (1989). Colloidal Gold: Principles, Methods, and Applications.

[31] Smith G.A., Marquis H., Jones S., Johnston N.C., Portnoy D.A., Goldfine H. (1995). The two distinct phospholipases C of *Listeria monocytogenes* have overlapping roles in escape from a vacuole and cell-to-cell spread. Infect. Immun..

[32] Dramsi S., Cossart P. (2003). Listeriolysin O-mediated calcium influx potentiates entry of *Listeria monocytogenes* into the human Hep-2 epithelial cell line. Infect. Immun..

[33] Nygard Skalman L., Holst M.R., Larsson E., Lundmark R. (2018). Plasma membrane damage caused by listeriolysin O is not repaired through endocytosis of the membrane pore. Biol. Open.

[34] Cajnko M.M., Marušić M., Kisovec M., Rojko N., Benčina M., Caserman S., Anderluh G. (2015). Listeriolysin O Affects the Permeability of Caco-2 Monolayer in a Pore-Dependent and Ca2+-Independent Manner. PLoS ONE.

[35] Repp H., Pamukci Z., Koschinski A., Domann E., Darji A., Birringer J., Brockmeier D., Chakraborty T., Dreyer F. (2002). Listeriolysin of *Listeria monocytogenes* forms Ca2+-permeable pores leading to intracellular Ca2+ oscillations. Cell. Microbiol..

[36] Stachowiak R., Łyżniak M., Grabowska M., Roeske K., Jagielski T., Bielecki J., Budziszewska B.K., Hoser G., Kawiak J. (2014). Cytotoxicity of purified listeriolysin O on mouse and human leukocytes and leukaemia cells. BMC Biotechnol..

[37] Sun R., Liu Y. (2013). Listeriolysin O as a strong immunogenic molecule for the development of new anti-tumor vaccines. Hum. Vaccines Immunother..

[38] Zhang T., Bae D., Wang C. (2015). Listeriolysin O mediates cytotoxicity against human brain microvascular endothelial cells. FEMS Microbiol. Lett..

[39] Gatenby R.A., Gillies R.J. (2004). Why do cancers have high aerobic glycolysis?. Nat. Rev. Cancer.

[40] Warburg O. (1956). On the origin of cancer cells. Science.

[41] Massari F., Ciccarese C., Santoni M., Iacovelli R., Mazzucchelli R., Piva F., Scarpelli M., Berardi R., Tortora G., Lopez-Beltran A. (2016). Metabolic phenotype of bladder cancer. Cancer Treat. Rev..

[42] Swietach P., Vaughan-Jones R.D., Harris A.L. (2007). Regulation of tumor pH and the role of carbonic anhydrase 9. Cancer Metastasis Rev..

[43] Fischer K., Hoffmann P., Voelkl S., Meidenbauer N., Ammer J., Edinger M., Gottfried E., Schwarz S., Rothe G., Hoves S. (2007). Inhibitory effect of tumor cell-derived lactic acid on human T cells. Blood.

[44] Visnjar T., Romih R., Zupancic D. (2019). Lectins as possible tools for improved urinary bladder cancer management. Glycobiology.

[45] Zupancic D., Kreft M.E., Romih R. (2014). Selective binding of lectins to normal and neoplastic urothelium in rat and mouse bladder carcinogenesis models. Protoplasma.

[46] Shewell L.K., Day C.J., Jen F.E., Haselhorst T., Atack J.M., Reijneveld J.F., Everest-Dass A., James D.B.A., Boguslawski K.M., Brouwer S. (2020). All major cholesterol-dependent cytolysins use glycans as cellular receptors. Sci. Adv..

[47] Chen H., Konofagou E.E. (2014). The size of blood-brain barrier opening induced by focused ultrasound is dictated by the acoustic pressure. J. Cereb. Blood Flow Metab..

[48] Atanassoff A.P., Wolfmeier H., Schoenauer R., Hostettler A., Ring A., Draeger A., Babiychuk E.B. (2014). Microvesicle shedding and lysosomal repair fulfill divergent cellular needs during the repair of streptolysin O-induced plasmalemmal damage. PLoS ONE.

[49] Wolfmeier H., Radecke J., Schoenauer R., Koeffel R., Babiychuk V.S., Drucker P., Hathaway L.J., Mitchell T.J., Zuber B., Draeger A. (2016). Active release of pneumolysin prepores and pores by mammalian cells undergoing a *Streptococcus pneumoniae* attack. Biochim. Biophys. Acta.

[50] Keyel P.A., Loultcheva L., Roth R., Salter R.D., Watkins S.C., Yokoyama W.M., Heuser J.E. (2011). Streptolysin O clearance through sequestration into blebs that bud passively from the plasma membrane. J. Cell Sci..

[51] Monastyrskaya K., Babiychuk E.B., Draeger A., Burkhard F.C. (2013). Down-regulation of annexin A1 in the urothelium decreases cell survival after bacterial toxin exposure. J. Urol..

[52] Coelho C., Brown L., Maryam M., Vij R., Smith D.F.Q., Burnet M.C., Kyle J.E., Heyman H.M., Ramirez J., Prados-Rosales R. (2019). virulence factors, including listeriolysin O, are secreted in biologically active extracellular vesicles. J. Biol. Chem..

[53] Višnjar T., Chesi G., Iacobacci S., Polishchuk E., Resnik N., Robenek H., Kreft M., Romih R., Polishchuk R., Kreft M.E. (2017). Uroplakin traffic through the Golgi apparatus induces its fragmentation: New insights from novel in vitro models. Sci. Rep..

[54] Cho I.C., Kim E.K., Joung J.Y., Seo H.K., Chung J., Park W.S., Lee K.H. (2012). Adjuvant intravesical instillation for primary T1G3 bladder cancer: BCG versus MMC in Korea. Anticancer Res..

[55] Smith J.A., Labasky R.F., Cockett A.T.K., Fracchia J.A., Montie J.E., Rowland R.G. (1999). Bladder cancer clinical guidelines panel summary report on the management of nonmuscle invasive bladder cancer (stages Ta, T1 and TIS). J. Urol..

