# Supplementary material for: Cytotoxic Activity of LLO Y406A Is Targeted to the Plasma Membrane of Cancer Urothelial Cells
Source: Int J Mol Sci. 2021 Mar 24;22(7):3305. doi: 10.3390/ijms22073305 (PMC8037347; doi:10.3390/ijms22073305)
Supplement: Supplementary file 1 [file ijms-22-03305-s001.pdf]

Supplementary Figure 1

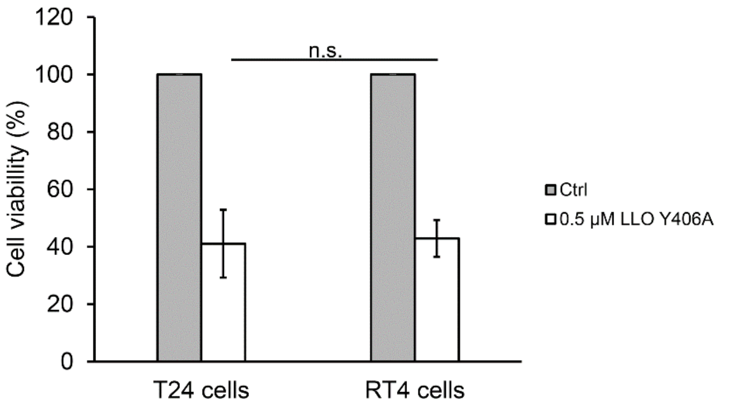

Supplementary Figure S1: Comparison in the viability of T24 and RT4 cells after 0.5 µM LLO Y406A treatment. Presented are averages ± SEM of three independent measurements, measured in triplicates.

Supplementary Figure 2

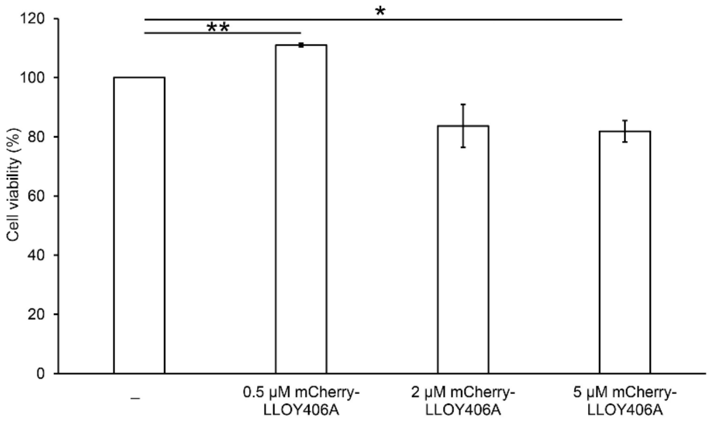

Supplementary Figure S2: The viability of RT4 cells after mCherry-LLO Y406A treatment. Cells were treated with 0.5, 2 and 5 µM mCherry-LLO Y406A for 2 hours, or were untreated (Control). One hour after treatment viabilities were measured. Presented are averages ± SEM of three independent measurements, measured in triplicates.

Supplementary Figure S3

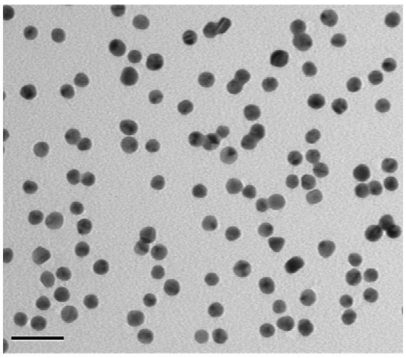

Supplementary Figure S3: Transmission electron microscopy of gold NPs. Gold NPs have an average diameter 10 ± 2nm. Scale bar is 50 nm.
